# Behind the Scenes: The Impact of Bioactive Phenylpropanoids on the Growth Phenotypes of Arabidopsis Lignin Mutants

**DOI:** 10.3389/fpls.2021.734070

**Published:** 2021-09-09

**Authors:** Ilias El Houari, Wout Boerjan, Bartel Vanholme

**Affiliations:** ^1^Department of Plant Biotechnology and Bioinformatics, Ghent University, Ghent, Belgium; ^2^VIB Center for Plant Systems Biology, Ghent, Belgium

**Keywords:** salicylic acid, cinnamic acid, flavonoids, ferulic acid, DCG, CINNAMATE-4-HYDROXYLASE, HYDROXYCINNAMOYL-CoA:SHIKIMATE HYDROXYCINNAMOYL TRANSFERASE, *p*-COUMAROYL SHIKIMATE/QUINATE 3′-HYDROXYLASE

## Abstract

The phenylpropanoid pathway converts the aromatic amino acid phenylalanine into a wide range of secondary metabolites. Most of the carbon entering the pathway incorporates into the building blocks of lignin, an aromatic polymer providing mechanical strength to plants. Several intermediates in the phenylpropanoid pathway serve as precursors for distinct classes of metabolites that branch out from the core pathway. Untangling this metabolic network in Arabidopsis was largely done using phenylpropanoid pathway mutants, all with different degrees of lignin depletion and associated growth defects. The phenotypic defects of some phenylpropanoid pathway mutants have been attributed to differentially accumulating phenylpropanoids or phenylpropanoid-derived compounds. In this perspectives article, we summarize and discuss the reports describing an altered accumulation of these bioactive molecules as the causal factor for the phenotypes of lignin mutants in Arabidopsis.

## Introduction

**FIGURE 1:**
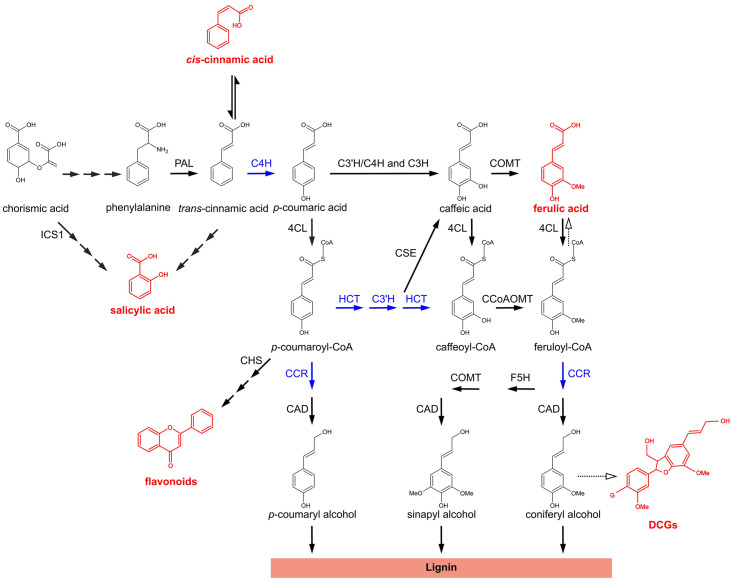
The phenylpropanoid pathway in *Arabidopsis thaliana*. Metabolic pathway showing the biosynthesis of different classes of phenylpropanoids and phenylpropanoid-derived compounds. For flavonoids and DCGs a molecular structure is shown representative for their respective class of compounds. Metabolites and metabolite classes shown in red are discussed in this work for their (putative) role in the phenotypes of lignin mutants. Blue arrows indicate enzymatic steps upon which perturbation leads to the accumulation of bioactive molecules that have been proposed to be implicated in dwarfing of lignin mutants; solid arrows represent enzymatic steps validated by experimental evidence; dashed arrows represent suggested enzymatic steps/pathways; two or more successive smaller solid arrows represent multiple enzymatic steps. PAL, PHENYLALANINE AMMONIA-LYASE; C4H, CINNAMATE 4-HYDROXYLASE; 4CL, 4-COUMARATE:CoA LIGASE; HCT, *p*-HYDROXYCINNAMOYL-CoA:QUINATE/SHIKIMATE *p*-HYDROXYCINNAMOYLTRANSFERASE; C3′H, *p*-COUMAROYL SHIKIMATE/QUINATE 3′-HYDROXYLASE; C3H, *p*-COUMARATE 3-HYDROXYLASE; CSE, CAFFEOYL SHIKIMATE ESTERASE; CCoAOMT, CAFFEOYL-CoA *O*-METHYLTRANSFERASE; CCR, CINNAMOYL-CoA REDUCTASE; F5H, FERULATE 5-HYDROXYLASE; COMT, CAFFEATE *O*-METHYLTRANSFERASE; CAD, CINNAMYL ALCOHOL DEHYDROGENASE; ICS1, ISOCHORISMATE SYNTHASE 1; CHS, CHALCONE SYNTHASE; DCGs, dehydrodiconiferyl alcohol glucosides; G, glucose.

The general phenylpropanoid pathway (PPP) is a central metabolic pathway in plants involved in the synthesis of a broad range of secondary metabolites that consist of aromatic ring structures with particular sidechain modifications (Vogt, 2010; Figure 1). The first enzyme of the pathway, PHENYLALANINE AMMONIA LYASE (PAL) deaminates the aromatic amino acid phenylalanine. This results in the formation of *trans*-cinnamic acid (*t*-CA), which is subsequently converted to *p*-coumaric acid by CINNAMATE-4-HYDROXYLASE (C4H). Next, 4-HYDROXYCINNAMATE-CoA LIGASE (4CL) converts *p*-coumaric acid to *p*-coumaroyl-CoA, which is subsequently converted to *p*-coumaroyl-shikimate by HYDROXYCINNAMOYL-CoA:SHIKIMATE HYDROXYCINNAMOYL TRANSFERASE (HCT). *p*-COUMAROYL SHIKIMATE/QUINATE 3′-HYDROXYLASE (C3′H) hydroxylates the shikimate conjugate and the product of this reaction, caffeoyl shikimate, is subsequently converted to caffeoyl-CoA by HCT. Caffeoyl-CoA is further converted to feruloyl-CoA by CAFFEOYL-CoA *O*-METHYLTRANSFERASE (CCoAOMT). *p*-Coumarate can also be shuttled directly to caffeate by either *p*-COUMARATE 3-HYDROXYLASE (C3H or APX1; Barros et al., 2019) or a C3′H/C4H enzyme complex (Chen et al., 2011). Additionally, the second conversion by HCT can be bypassed by CAFFEOYL SHIKIMATE ESTERASE (CSE), converting caffeoyl shikimate to caffeate and 4CL, converting caffeate to caffeoyl-CoA.

Most carbon skeletons entering the PPP find their way to the monolignol biosynthesis pathway, hereby leading to the building blocks of lignin (Boerjan et al., 2003), a highly recalcitrant polymer mainly deposited in the secondary-thickened plant cell wall, where it provides mechanical strength and hydrophobicity. *p*-Coumaryl alcohol and coniferyl alcohol are produced *via* the concerted actions of CINNAMOYL-CoA REDUCTASE (CCR) and CINNAMYL ALCOHOL DEHYDROGENASE (CAD), whereas sinapyl alcohol requires an additional FERULATE 5-HYDROXYLASE (F5H) and CAFFEATE *O*-METHYLTRANSFERASE (COMT) step. In addition to serving for monolignol biosynthesis, several intermediates of the PPP serve as branch points toward other pathways leading to the production of a wide range of secondary metabolites such as flavonoids, coumarins, and benzoic acids (Vogt, 2010).

The central role of the PPP in plant secondary metabolism implies its involvement in a plethora of processes. Genetic or chemical perturbations therefore often come with a variety of phenotypic defects (Ha et al., 2021) of which the severity is frequently linked to the degree of perturbation and the level of genetic redundancy. The different phenotypes range from overall dwarfism to more distinct phenotypes such as increased or decreased lateral rooting, increased shoot branching, male sterility, and a decreased seed set. Several hypotheses have been proposed that explain these PPP mutant phenotypes in Arabidopsis and for a more elaborate description and graphical depiction of these hypotheses we refer to the review by Muro-Villanueva et al. (2019). In summary, two models suggest a depletion in lignin to lie at the basis of the observed dwarfism, also known as lignin modification-induced dwarfism (LMID; Muro-Villanueva et al., 2019). One proposes a lowered lignin content to cause a loss of mechanical strength or hydrophobicity in the xylem. The other model suggests a shift in cell wall integrity to trigger a stress response, resulting in growth defects. Besides these two models, a third proposes the differential accumulation of soluble pathway intermediates or derivatives thereof as the causal factor of the growth phenotypes. We will define this here as soluble phenylpropanoid-induced dwarfism (SPID). Despite the evidence for this third model, there is significant discussion on this topic, as in several cases initial findings could not be confirmed or were refuted. Here, we critically review and provide our perspectives on SPID in Arabidopsis.

## Accumulating Bioactive Molecules Causing Growth Defects in Arabidopsis Lignin Mutants

The extensive conjugation and detoxification of accumulating phenylpropanoids and phenylpropanoid-derived compounds in Arabidopsis (Vanholme R. et al., 2019) suggest that several of these compounds are bioactive. Correspondingly, the literature is scattered with studies claiming evidence for bioactivity of nearly every intermediate or derivative of the PPP on plant growth and development (Vanholme B. et al., 2019). Many of these studies should, however, be interpreted with care as they were never subjected to rigorous independent scrutiny and only a handful of these studies remains significant when taking physiological relevance in mind. In this context, a bioactive molecule is defined as a compound of natural origin that triggers a measurable biological effect at a concentration reflecting the endogenous concentration while taking stability, uptake, transport, and metabolization into consideration (Vanholme B. et al., 2019). Under this definition, several phenylpropanoids and phenylpropanoid-derived compounds have well-described bioactive properties in plants. For several of these compounds their differential accumulation has also been reported to cause PPP mutant phenotypes in Arabidopsis. These reports will be discussed below.

### Salicylic Acid

Salicylic acid (SA) is closely linked to the PPP and known to be an important signaling compound in plant defense responses against both biotic and abiotic stresses (Fragnière et al., 2011). This combination makes SA a likely candidate to explain some of the growth defects observed in lignin mutants. Production of SA is facilitated *via* two different routes (Lefevere et al., 2020) of which one branches directly from the PPP, more specifically from the intermediate *t*-CA (Richmond and Bleecker, 1999; Figure 1). A second route, which produces most of the SA in Arabidopsis, goes *via* the production of isochorismate through the shikimate pathway (Wildermuth et al., 2001; Torrens-Spence et al., 2019) and is positioned upstream of the PPP (Figure 1). Possibly as a consequence of the position of these pathways, either upstream or at the entry point of the PPP, SA typically accumulates when the PPP is blocked downstream of PAL (Schoch et al., 2002). For example, an increase in SA was observed in a dwarfed *HCT*-RNAi line (Gallego-Giraldo et al., 2011a). Blocking SA accumulation in these mutants by crossing them with either an *isochorismate synthase 1* mutant (*sid2*; Figure 1) deficient in SA biosynthesis or the SA-conjugating *NahG* line partially restored plant growth, indicating an involvement of SA in the induced dwarfism of lignin mutants. Evidence was provided that a mediation of gibberellic acid signaling might be involved in the SA-induced dwarfism (Gallego-Giraldo et al., 2011a), although the exact mechanism is still unclear.

In concordance with the *HCT*-RNAi line, SA levels were increased in a *c3*′*h* mutant (*ref8-1*) (Kim et al., 2014) and restoration of the pathway by reintroducing *C3*′*H* expression restored growth and brought SA content back to WT levels. However, in contrast to the *HCT*-RNAi line, preventing SA accumulation in the *ref8-1* mutant by crossing it with the *NahG* line did not result in growth restoration. A parallel study wherein the *ref8-1* mutant was crossed with a *sid2* mutant also showed SA accumulation not to be at the basis of the growth phenotypes (Bonawitz et al., 2014). A *ref8-1 mediator* (*med*)*5a/5b* double mutant, however, showed a full growth restoration while still having increased levels of SA. It is striking that SA would cause growth reduction of *HCT*-downregulated plants but not that of *c3*′*h* mutants, given their proximal position in the PPP and their comparable mutant phenotypes. Therefore, whereas SA accumulation seems to be at the basis of dwarfism of *HCT*-downregulated plants, the results obtained for the *c3*′*h* mutant question the role of SA accumulation as a general mechanism underlying growth reduction in PPP mutants.

### Cinnamic Acid

Cinnamic acid (CA) is produced upon deamination of phenylalanine by PAL (Figure 1). Although the enzymatic reaction results in the formation of *t*-CA, which is further metabolized by the PPP, also its bioactive *cis*-isomer (*c*-CA) is present in plants (Yin et al., 2003; Wong et al., 2005; El Houari et al., 2021). Both isomers are readily interconvertible under the influence of UV-light, and this mechanism has long been considered as a source for *c*-CA production (Yin et al., 2003; Wong et al., 2005). Recently, evidence was also provided for a UV-independent and possibly dedicated enzymatic biosynthesis of *c*-CA in plants (El Houari et al., 2021). The possibility of accumulating CA-esters or their derivatives having a role in the observed growth perturbation of PPP mutants was already coined by Franke et al. (2002) and CA has indeed been implicated in the growth defects found in several *c4h* mutants (Schilmiller et al., 2009; Kurepa et al., 2018; Kurepa and Smalle, 2019; El Houari et al., 2021). Genetic studies toward *C4H* were mainly performed on weak *c4h* mutants (*ref3-1*; *ref3-2*; *ref3-3*; Schilmiller et al., 2009) nonetheless showing large changes in lignin content and composition. The depletion in lignin went paired with dwarfism, sterility, and increased branching (Schilmiller et al., 2009). However, it was later suggested that the increased branching of the *ref3-1* mutant, as well as a then-observed increase in lateral rooting, were the result of a altered auxin sensitivity caused by the accumulation of a PPP intermediate upstream of C4H (Kurepa et al., 2018). Subsequent investigation indicated the responsible bioactive compound to be downstream of PAL. Specifically, *t*-CA or a *t*-CA derivative was put forward as responsible for the increased branching and lateral rooting. Another allelic mutant, *ref3-3*, showed an increase in rosette size and biomass (Kurepa and Smalle, 2019). This growth increase was again suggested to be caused by an upstream accumulation of *t*-CA, as exogenous application of *t*-CA to wild-type plants facilitated a similar growth-promotion (Kurepa and Smalle, 2019).

Schilmiller et al. (2009) reported local swellings in the branch junctions of the stems of the *ref3-1* mutant and coined accumulating *c*-CA as being responsible. In agreement with these findings, a later study showed *c*-CA rather than *t*-CA to be the bioactive isoform of CA (Steenackers et al., 2017). Supplementing plants with *c*-CA inhibits auxin transport in the root tip of the plant, causing a local build-up in auxin concentrations, which results in strong proliferation of lateral rooting. In addition, a follow-up study showed *c*-CA and not *t*-CA to facilitate growth-promotion upon exogenous application (Steenackers et al., 2019). By preventing isomerization using light conditions devoid of UV or using conformationally constrained phenylcyclopropanoid analogs of both CA isomers, *c*-CA was shown to be the growth-promoting isomer. Finally, increased levels of *c*-CA were recently indicated to block auxin transport in the hypocotyl of Arabidopsis seedlings upon inhibition of C4H. This lead to a local proliferation of adventitious roots in the upper part of the hypocotyl (El Houari et al., 2021). These observations on the bioactive properties of *c*-CA are in line with the observed increased lateral rooting and branching of the *ref3-1* mutant and the increased rosette size of the *ref3-3* mutant. This thus suggests that the accumulation of *c*-CA and not *t*-CA lies at the basis of these phenotypes.

### Flavonoids

The flavonoid biosynthetic pathway branches from *p*-coumaroyl-CoA and further steps result in the production of a wide range of metabolites, including flavones, flavonols, anthocyanins, chalcones, and flavan-3-ols (Lepiniec et al., 2006). These all share a common backbone consisting of two phenyl rings and one heterocyclic ring (Figure 1) and are involved in a range of processes in the plant, including plant defense (Treutter, 2005), oxidative stress responses (Nakabayashi et al., 2014), nodulation (Kobayashi et al., 2004), and pigmentation (De Jong et al., 2004; Eichhorn and Winterhalter, 2005). Flavonoids have also been coined to steer plant development *via* two mechanisms. Firstly, flavonoids have antioxidative properties (Agati et al., 2012) and impaired biosynthesis of flavonoids results in higher reactive oxygen species (ROS) levels in the plant (Watkins et al., 2014; Gayomba and Muday, 2020). For example, the flavonoid kaempferol was described as a negative regulator of lateral root growth, most likely by regulating ROS levels in the lateral root primordia (Chapman and Muday, 2021). Secondly, the flavonoids naringenin, quercetin, and kaempferol have also been described to be auxin transport inhibitors (Jacobs and Rubery, 1988; Brunn et al., 1992; Faulkner and Rubery, 1992) and endogenous over- or underproduction of flavonoids was shown to influence auxin transport using mutants in the flavonoid biosynthetic pathway (Murphy et al., 2000; Brown et al., 2001; Peer et al., 2004; Buer et al., 2013). An *hct* mutant with severe growth reduction and reduced leaf size showed an apparent increase in flavonoid levels, as the leaves of this mutant showed a purple coloration (Hoffmann et al., 2004). Such an accumulation of flavonoids, anthocyanins, and other flavonoid derivatives upon blocking HCT was later confirmed in *HCT*-RNAi plants (HCT^–^) (Besseau et al., 2007) and reducing flavonoid levels by growing the HCT^–^ plants under low-light conditions was correlated to a restoration in growth. Additionally, reducing flavonoid content in HCT^–^ plants by crossing them with flavonoid-deficient *CHS*-RNAi plants (CHS^–^; Figure 1) also correlated with a growth restoration. Both the increased flavonoid content and growth inhibition coincided with an inhibition in auxin transport in these plants. A later study, however, indicated that silencing of *HCT* in a *chs* knockout (*tt4*) background deficient for flavonoids did inhibit plant growth (Li et al., 2010). In fact, the degree of growth inhibition upon silencing *HCT* in the *tt4* background was similar to that upon silencing *HCT* in WT plants. This indicated that the observed growth inhibition of the HCT^–^ plants was most likely not due to the accumulation of flavonoids. Notably, the growth restoration in the double silenced HCT^–^/CHS^–^ plants did go paired with a slight restoration in lignification. This is most likely due to promoter silencing of the *HCT*-RNAi construct, as both *HCT* and *CHS* RNAi constructs were driven by a *35S* promoter (Li et al., 2010). The partial growth restoration of the HCT^–^/CHS^–^ plants could thus find its origin in the slight restoration in lignification of these plants rather than the reduction in flavonoid content. Moreover, repetition of flavonoid quantification indicated that the total amount of flavonoids per rosette is the same for WT and HCT^–^ plants. Similarly, a CRISPR-generated *hct* mutant (*hct*^*D7*^; Kriegshauser et al., 2021) showed an 80-fold increase in levels of *p*-coumaroyl-glucose but not in kaempferol 3-*O*-rhamnoside 7-*O*-rhamnoside, indicating that also here flavonoid levels are not significantly altered when blocking HCT. In summary, flavonoid accumulation does not seem to be responsible for the dwarfism of HCT^–^ plants. Therefore, whereas altered flavonoid content does modulate auxin transport in flavonoid biosynthesis mutants and possibly also in the HCT^–^ plants, there is no conclusive evidence that their differential accumulation in lignin mutants causes dwarfism.

### Ferulic Acid

Ferulic acid is an intermediate of the PPP, being produced from caffeic acid by COMT (Figure 1). Like other phenylpropanoids it has known antioxidant properties and ROS scavenging potential (Graf, 1992; Kanski et al., 2002). Whereas many studies have investigated the bioactive properties of ferulic acid (Lippincott and Lippincott, 1971; Li et al., 1993; Locher et al., 1994), convincing evidence for a bioactive role in plants is scarce. In *ccr1* mutants, ferulate conjugate levels accumulate and higher levels of ferulic acid are incorporated in the lignin (Derikvand et al., 2008; Vanholme et al., 2012; De Meester et al., 2018). The accumulation of ferulic acid was found to be correlated to growth phenotypes in *ccr1-4* plants (Xue et al., 2015), and its antioxidant potential was proposed to bring about a reduced leaf size, as the *ccr1-4* cells remained longer in a mitotic state. This delay in cell proliferation exit resulted in plants with a higher cell count but smaller cell size, causing a reduction in overall leaf size. ROS are known to be required for the shift of cells toward cell proliferation exit (Boonstra and Post, 2004; Tsukagoshi et al., 2010). The accumulation of ferulic acid was therefore proposed to bring about the scavenging of ROS through its antioxidant action, hereby being at the basis of the delay in cell proliferation exit. A later study, however, found dwarfism in a *ccr1-6* mutant to be fully restored upon restoration of lignin specifically in the xylem vessels, despite increased levels of ferulic acid coupling products in the leaves (De Meester et al., 2018). In addition, the reduced cell proliferation exit in the vessel-complemented *ccr1-6* plants was mitigated, indicating (1) that ferulic acid is not at the basis of the dwarfism, and (2) that ferulic acid is not at the basis of the observed delay in cell proliferation exit.

### Dehydrodiconiferyl Alcohol Glucosides

Dehydrodiconiferyl alcohol glucosides or DCGs form a specific class of glucosylated phenylpropanoid coupling products with proposed hormone-like activity. The DCG aglycon is a coniferyl alcohol dimer, coupled *via* a coumaran linkage (Figure 1). Coupling of the two coniferyl alcohol radicals leads to two chiral centers in the molecule, resulting in different stereoisomers. Interestingly, the bioactivity of DCGs is restricted to particular diastereoisomers that were initially isolated from tumor cells of *Vinca rosea* (Lynn et al., 1987) and several follow up studies described the accumulation of DCGs in rapidly dividing tissues or cell cultures (Binns et al., 1987; Attoumbré et al., 2006; Hano et al., 2006). In tobacco tissue culture, DCGs were also able to replace cytokinin in cell division assays (Binns et al., 1987) and cytokinin treatment effectively stimulated DCG accumulation (Teutonico et al., 1991), suggesting that DCG biosynthesis in the plant is controlled by cytokinin. Based on these observations, these molecules have been described as having cell division-promoting activities (Binns et al., 1987; Lynn et al., 1987; Teutonico et al., 1991). As they are formed from coniferyl alcohol, positioned at the final step of the PPP (Figure 1), DCG concentrations are often lowered in PPP mutants (Vanholme et al., 2012; Dima et al., 2015). Together with their possible role in the stimulation of plant cell division, this depletion is frequently used to explain the dwarfism of Arabidopsis PPP mutants (Franke et al., 2002; Abdulrazzak et al., 2006; Do et al., 2007; Li et al., 2010). However, a causal role for DCG depletion in PPP mutant dwarfism has not yet been shown. Moreover, feeding the probable DCG precursor coniferyl alcohol to *Nicotiana benthamiana* seedlings did severely impair instead of stimulate growth, although this could also likely be the result of an induced lignification (Väisänen et al., 2015). In addition, to our knowledge no further physiological support for the cell division promoting activity by DCGs has been provided since the initial reports over 30 years ago (Binns et al., 1987; Lynn et al., 1987; Teutonico et al., 1991). Together, the involvement of DCGs in PPP mutant dwarfism thus remains purely speculative.

## The Role of Bioactive Phenylpropanoids in PPP Mutant Phenotypes Is Underexplored

A large number of studies report on either the phenotype of PPP mutants or on the bioactive properties of phenylpropanoids or phenylpropanoid-derived compounds. In comparison, however, the available evidence for SPID is minor. In addition, the evidence that should support SPID has often been contested. This seems to suggest that the accumulation of bioactive phenylpropanoids rarely results in observable phenotypes in PPP mutants and that lignin depletion is the predominant and in most cases only factor causing growth defects. Whereas it is reasonable to assume that LMID is a main cause of dwarfism in PPP mutants considering the vast number of such reports, it seems precarious to assume that SPID is negligible, and that differential accumulation of phenylpropanoids and phenylpropanoid-derived compounds would not induce any phenotypic alterations or defects in the plant.

The investigation on SPID comes with some difficulties that could explain the low number of these studies. The depletion in lignin is a trait commonly observed in PPP mutants, as lignin monomers are end products of the pathway. Mutation and inhibition of each step of the pathway will therefore often result in a lowered lignin content. Each PPP mutant will, however, accumulate a different set of intermediates depending on the position of the inhibited step in the PPP. This is exemplified by, e.g., *hct* and *c4h* mutants. Both have lower lignin levels, but the former also accumulates anthocyanins, resulting in a purple coloration (Hoffmann et al., 2004). Similar coloration is not observed in the *c4h* mutants, as flavonoid production (including anthocyanins) lies downstream of C4H. In addition, each class of phenylpropanoids may harbor a set of different bioactive properties that may or may not yet be fully elucidated. The unique alterations in metabolic flux upon mutations in each step of the pathway coupled with the distinct bioactive properties that each class of phenylpropanoids may harbor encumbers inquiries toward their involvement in possible phenotypes.

Because lignin depletion in the cell wall by itself is often responsible for a large portion of the phenotype, more subtle phenotypes induced by accumulating bioactive compounds might go unnoticed. In addition, SPID can be overlooked due to the implemented experimental conditions. For example, flavonoids are known UV protectants, and their production in the plant increases upon exposure to a higher light intensity (Jaakola et al., 2004; Tattini et al., 2004). In accordance, low light conditions attenuated the flavonoid accumulation in *HCT*-silenced plants (Besseau et al., 2007). Also, phenotypic defects caused by *c*-CA were effectively mitigated by growing plants under UV-free light (El Houari et al., 2021). As flux through the phenylpropanoid pathway is regulated by a plethora of environmental conditions such as light intensity, temperature, and abiotic stresses (Christie et al., 1994; Hemm et al., 2004; Sharma et al., 2019), it is likely that certain phenotypes go unnoticed under certain experimental conditions.

## Perspectives and Outlook

Considering the pathway’s complexity and the vast changes in metabolic flux upon its inhibition it seems unlikely that lignin depletion is the only causative agent of PPP mutant phenotypes (Vanholme et al., 2012). Indeed, restoration of lignification upon perturbation of the PPP does not always come paired with a total restoration in plant growth (Kim et al., 2014; El Houari et al., 2021), indicating that there are still other factors at play in the observed growth defects. Moreover, it is likely that of the different proposed models explaining dwarfism in PPP mutants (Muro-Villanueva et al., 2019) several are true for the same mutant, as recently evidenced upon blocking C4H (El Houari et al., 2021). Chemical inhibition of C4H study resulted in the accumulation of adventitious roots apically in the hypocotyl. This phenotype was caused jointly by an upstream increase in *c*-CA and a downstream depletion in lignin, thus showing that both an accumulation of bioactive intermediates and downstream depletion in lignin can result in phenotypes within the same mutant.

Arabidopsis may not be sufficient as the only model system to investigate the role of SPID in PPP mutant phenotypes. The general role of lignin is similar in all vascular plant species, as it confers rigidity and hydrophobicity to the secondary cell wall. In contrast, the bioactivity of PPP intermediates can differ in both nature and strength between different species. For example, flavonoid levels are increased in *hct* mutants in both alfalfa (*Medicago sativa*) and Arabidopsis (Besseau et al., 2007; Gallego-Giraldo et al., 2011b). However, only in Arabidopsis did the increase in flavonoids go paired with a reduction in auxin transport. This indicates that phenotypic defects caused by accumulating bioactive phenylpropanoids can be species-specific and dependent on the genetic makeup of the plant. In addition, the inhibitory strength of *c*-CA on root growth was markedly different between monocots and dicots (Steenackers et al., 2017). This could be due to differences in uptake, detoxification and metabolization of *c*-CA. An alternative explanation for this difference may also be found in the target-specificity of the compound. *c*-CA targets auxin transporters or other members of the auxin transport machinery (Steenackers et al., 2017), cis-Cinnamic acid is a novel, natural auxin efflux inhibitor that promotes lateral root formation. Both the number and protein sequences of auxin transporters strongly differ between species (Carraro et al., 2012; Yang et al., 2019), thus providing a possible explanation to the difference in inhibitory strength. In addition, the differences in endogenous concentrations of bioactive compounds between species could also play a role. For example, the basal levels in SA are markedly higher in rice as compared to Arabidopsis (Yang et al., 2004). An increase in absolute levels of SA in rice would therefore make less of an impact on plant growth as compared to Arabidopsis. These hypotheses could thus provide an explanation for some of the differences in phenotype when knocking out the same gene of the PPP in different species. For example, knocking out *CSE* causes a much stronger growth perturbation in *Medicago truncatula* as compared to Arabidopsis (Vanholme et al., 2013; Ha et al., 2016). It is possible that accumulation the substrate of CSE, caffeoyl shikimate, or any of the other upstream accumulating molecules has stronger growth inhibitory effects in Medicago than in Arabidopsis. However, it also needs to be said that the measured lignin content was significantly lower in Medicago than in Arabidopsis (over 80% as compared to 36%, respectively; Vanholme et al., 2013; Ha et al., 2016), which could thus also explain for the difference in phenotype.

Investigation on SPID thus comes with certain difficulties. It is, however, essential to understand the molecular mechanisms underpinning SPID to allow for the engineering of plants with improved biomass quality while mitigating a yield penalty. Toward this end, elucidating the mode of action of bioactive phenylpropanoids is crucial, as this allows a pinpointed investigation toward their involvement in the observed phenotypes.

## Data Availability Statement

The original contributions presented in the study are included in the article/supplementary material, further inquiries can be directed to the corresponding author.

## Author Contributions

All authors listed have made a substantial, direct and intellectual contribution to the work, and approved it for publication.

## Conflict of Interest

The authors declare that the research was conducted in the absence of any commercial or financial relationships that could be construed as a potential conflict of interest.

## Publisher’s Note

All claims expressed in this article are solely those of the authors and do not necessarily represent those of their affiliated organizations, or those of the publisher, the editors and the reviewers. Any product that may be evaluated in this article, or claim that may be made by its manufacturer, is not guaranteed or endorsed by the publisher.
